# (5,5′-Dimethyl-2,2′-bipyridine-κ^2^ *N*,*N*′)(1-naphthyl­acetato-κ*O*)(1-naphthyl­acetato-κ^2^ *O*,*O*′)zinc hemihydrate

**DOI:** 10.1107/S1600536811013353

**Published:** 2011-04-16

**Authors:** Li-Li Ji, Jian-She Liu, Wen-Dong Song

**Affiliations:** a Environment Science and Engineering, Donghua University, Shanghai 200051, People’s Republic of China; b College of Science, Guangdong Ocean University, Zhanjiang 524088, People’s Republic of China

## Abstract

In the title compound, [Zn(C_12_H_9_O_2_)_2_(C_12_H_12_N_2_)]·0.5H_2_O, the water mol­ecule lies on a twofold rotation axis. The Zn^II^ atom is coordinated by three O atoms from two 1-naphthyl­acetate ligands, one monodentate and the other asymmetric bidentate chelate, and two N atoms from a 5,5′-dimethyl-2,2′-bipyridine ligand, giving an irregular environment. In the crystal, the complex mol­ecules are inter­linked through the water mol­ecule by O—H⋯O_carboxyl­ate_ hydrogen bonds, together with weak C—H⋯O and bipyridine ring π–π stacking inter­actions [ring centroid separation = 3.761 (2) Å], giving a two-dimensional network structure.

## Related literature

For background to self-assembly of supra­molecular architectures based on naphthyl­carboxyl­ate ligands, see: Kong *et al.* (2009 ▶); Li *et al.* (2009 ▶). The Zn—O distance in the second ligand [2.417 (3) Å] suggests a non-negligible (bidentate) inter­action, see: Guilera & Steed (1999 ▶).
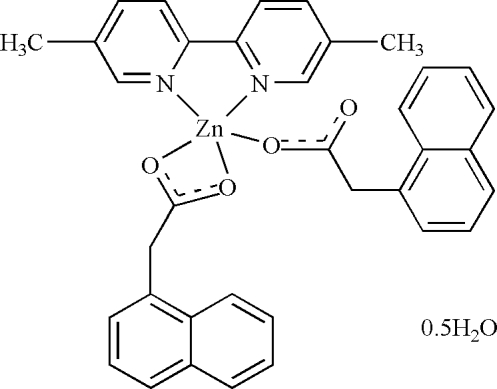


## Experimental

### 

#### Crystal data

[Zn(C_12_H_9_O_2_)_2_(C_12_H_12_N_2_)]·0.5H_2_O
*M* *_r_* = 629.00
Monoclinic, 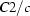

*a* = 32.212 (7) Å
*b* = 8.2668 (17) Å
*c* = 25.314 (5) Å
β = 117.865 (4)°
*V* = 5959 (2) Å^3^
*Z* = 8
Mo *K*α radiation
μ = 0.87 mm^−1^
*T* = 296 K
0.30 × 0.28 × 0.21 mm

#### Data collection

Bruker APEXII area-detector diffractometer
Absorption correction: multi-scan (*SADABS*; Sheldrick, 1996 ▶) *T*_min_ = 0.756, *T*_max_ = 0.819
21468 measured reflections
5325 independent reflections
3566 reflections with *I* > 2σ(*I*)
*R*_int_ = 0.049

#### Refinement

*R*[*F* ^2^ > 2σ(*F* ^2^)] = 0.044
*wR*(*F* ^2^) = 0.106
*S* = 0.99
5325 reflections
395 parameters
2 restraints
H-atom parameters constrained
Δρ_max_ = 0.45 e Å^−3^
Δρ_min_ = −0.30 e Å^−3^

### 

Data collection: *APEX2* (Bruker, 2004 ▶); cell refinement: *SAINT* (Bruker, 2004 ▶); data reduction: *SAINT*; program(s) used to solve structure: *SHELXS97* (Sheldrick, 2008 ▶); program(s) used to refine structure: *SHELXL97* (Sheldrick, 2008 ▶); molecular graphics: *SHELXTL* (Sheldrick, 2008 ▶); software used to prepare material for publication: *SHELXL97*.

## Supplementary Material

Crystal structure: contains datablocks I, global. DOI: 10.1107/S1600536811013353/zs2105sup1.cif

Structure factors: contains datablocks I. DOI: 10.1107/S1600536811013353/zs2105Isup2.hkl

Additional supplementary materials:  crystallographic information; 3D view; checkCIF report

## Figures and Tables

**Table 1:** Hydrogen-bond geometry (Å, °)

*D*—H⋯*A*	*D*—H	H⋯*A*	*D*⋯*A*	*D*—H⋯*A*
O5—H5⋯O1	0.82	2.21	2.948 (3)	150
C8—H8*A*⋯O4^i^	0.93	2.59	3.515 (6)	171

Symmetry code: (i) 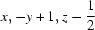.

**Fig. 1.**
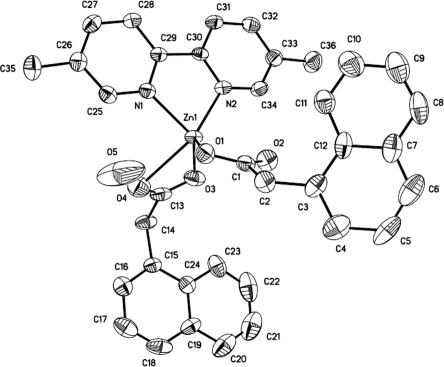
The structure of the title compound, showing the atom numbering scheme. The water molecule of solvation (O5) lies on a twofold rotation axis. Non-H atoms are shown with 30% probability displacement ellipsoids and H atoms are omitted.

**Fig. 2.**
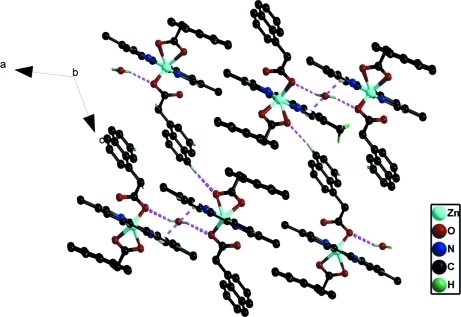
A view of the three-dimensional network in (I) showing O—H···O, C—H···O and π-π stacking interactions as dashed lines.

## supplementary crystallographic information

### Comment

Self-assembly of supramolecular architectures based on naphthylcarboxylate ligands has attracted much attention during recent decades (Kong *et al.*, 2009; Li *et al.*, 2009). However, to our knowledge, 1-naphthylacetic acid has not been used as a potential building block in the construction of supramolecular architectures.  Herein we report the structure of the title compound, the mixed ligand complex [(C_12_H_12_N_2_)(C_12_H_9_O_2_)_2_]_2_ . 0.5H_2_O (I), from the reaction of zinc nitrate with 1-naphthylacetic acid and 5,5'-dimethyl-2,2'-bipyridyrine in a basic aqueous solution.

The asymmetric unit in (I) (Fig. 1) consists of one Zn^II^ complex unit and a water molecule which lies on a two-fold rotation axis (Fig. 1). The five-coordinate Zn centre comprises three O atoms  from two 1-naphthylacetate ligands and two N atoms from a 5,5'-dimethyl-2,2'-bipyridyrine ligand. There are two coordination modes for the 1-naphthylacetate ligands in the structure: one monodentate the other asymmetric bidentate chelate. The Zn1-O4 distance in the second ligand [2.417 (3) Å] suggests a non-negligible (bidentate) interaction (Guilera & Steed, 1999) whereas the Zn1—O5 distance in the first ligand [2.587 (3)] is considered beyond the distance maximum for a bidentate interaction. In the crystal, the supramolecular network is stabilized by water O—H···O_carboxyl_, hydrogen bonds together with weak intermolecular aromatic C8—H···O_carboxyl_ interactions (Table 1), giving a two-dimensional network structure. In addition the inter-ring separation between the pyridine rings of two adjacent 5,5'-dimethyl-2,2'-bipyridine ligands is 3.761 (2) Å, indicating weak π-π stacking interactions (Fig. 2).

### Experimental

A mixture of 1-naphthylacetic acid (0.110 g, 0.5 mmol), 5,5'-dimethyl-2,2'-bipyridine (0.092 g, 0.5 mmol), zinc nitrate hexahydrate (0.075 g, 0.25 mmol), NaOH (0.08 g, 0.2 mmol) and water (10 ml) was placed in a 23 ml Teflon-lined reactor, which was heated to 423 K for 3 days, and then cooled to room temperature at a rate of 10 K h^-1^. The colorless crystals obtained were washed with water and dried in air (yield 47% based on zinc).

### Refinement

All H atoms were located from difference maps, and were treated as riding atoms with O—H = 0.82 Å and C—H = 0.93, 0.96 and 0.97 Å, for aryl, methyl and methine groups respectively, and with *U*_iso_(H) = 1.5*U*_eq_ (methyl C-atoms) and 1.2*U*_eq_(non-methyl C-atoms or water O-atom).

### Crystal data

**Table:**

[Zn(C_12_H_9_O_2_)_2_(C_12_H_12_N_2_)]·0.5H_2_O	*F*(000) = 2616
*M**_r_* = 629.00	*D*_x_ = 1.402 Mg m^−^^3^
Monoclinic, *C*2/*c*	Mo *K*α radiation, λ = 0.71073 Å
Hall symbol: -C 2yc	Cell parameters from 5837 reflections
*a* = 32.212 (7) Å	θ = 2.8–27.9°
*b* = 8.2668 (17) Å	µ = 0.87 mm^−^^1^
*c* = 25.314 (5) Å	*T* = 296 K
β = 117.865 (4)°	Block, colorless
*V* = 5959 (2) Å^3^	0.30 × 0.28 × 0.21 mm
*Z* = 8	

### Data collection

**Table:**

Bruker APEXII area-detector diffractometer	5325 independent reflections
Radiation source: fine-focus sealed tube	3566 reflections with *I* > 2σ(*I*)
graphite	*R*_int_ = 0.049
φ and ω scans	θ_max_ = 25.2°, θ_min_ = 2.6°
Absorption correction: multi-scan (*SADABS*; Sheldrick, 1996)	*h* = −38→38
*T*_min_ = 0.756, *T*_max_ = 0.819	*k* = −9→9
21468 measured reflections	*l* = −30→30

### Refinement

**Table:**

Refinement on *F*^2^	Primary atom site location: structure-invariant direct methods
Least-squares matrix: full	Secondary atom site location: difference Fourier map
*R*[*F*^2^ > 2σ(*F*^2^)] = 0.044	Hydrogen site location: inferred from neighbouring sites
*wR*(*F*^2^) = 0.106	H-atom parameters constrained
*S* = 0.99	*w* = 1/[σ^2^(*F*_o_^2^) + (0.0328*P*)^2^ + 11.176*P*] where *P* = (*F*_o_^2^ + 2*F*_c_^2^)/3
5325 reflections	(Δ/σ)_max_ = 0.020
395 parameters	Δρ_max_ = 0.45 e Å^−^^3^
2 restraints	Δρ_min_ = −0.30 e Å^−^^3^

### Special details

**Table:**

Geometry. All esds (except the esd in the dihedral angle between two l.s. planes) are estimated using the full covariance matrix. The cell esds are taken into account individually in the estimation of esds in distances, angles and torsion angles; correlations between esds in cell parameters are only used when they are defined by crystal symmetry. An approximate (isotropic) treatment of cell esds is used for estimating esds involving l.s. planes.
Refinement. Refinement of F^2^ against ALL reflections. The weighted R-factor wR and goodness of fit S are based on F^2^, conventional R-factors R are based on F, with F set to zero for negative F^2^. The threshold expression of F^2^ > 2sigma(F^2^) is used only for calculating R-factors(gt) etc. and is not relevant to the choice of reflections for refinement. R-factors based on F^2^ are statistically about twice as large as those based on F, and R- factors based on ALL data will be even larger.

### Fractional atomic coordinates and isotropic or equivalent isotropic displacement parameters (Å^2^)

**Table:**

	*x*	*y*	*z*	*U*_iso_*/*U*_eq_	
Zn1	0.120445 (14)	0.34832 (5)	0.280713 (17)	0.04660 (14)	
O1	0.07142 (9)	0.4782 (3)	0.21694 (10)	0.0619 (6)	
O2	0.12780 (10)	0.4724 (3)	0.19164 (11)	0.0687 (7)	
O3	0.18066 (9)	0.4490 (3)	0.34162 (11)	0.0691 (7)	
O4	0.12345 (12)	0.5188 (4)	0.35996 (13)	0.0879 (10)	
N1	0.08321 (9)	0.1801 (3)	0.30436 (11)	0.0448 (7)	
N2	0.14254 (9)	0.1318 (3)	0.26096 (11)	0.0442 (6)	
C1	0.08888 (14)	0.5173 (4)	0.18284 (15)	0.0533 (9)	
C2	0.05863 (14)	0.6281 (5)	0.13114 (16)	0.0660 (11)	
H2A	0.0269	0.5856	0.1120	0.079*	
H2B	0.0576	0.7335	0.1473	0.079*	
C3	0.07433 (12)	0.6504 (5)	0.08408 (17)	0.0647 (11)	
C4	0.09184 (16)	0.7942 (6)	0.0771 (2)	0.0952 (16)	
H4A	0.0946	0.8806	0.1021	0.114*	
C5	0.10634 (18)	0.8141 (8)	0.0303 (3)	0.107 (2)	
H5A	0.1182	0.9118	0.0249	0.129*	
C6	0.10167 (19)	0.6813 (9)	−0.0053 (3)	0.108 (2)	
H6A	0.1106	0.6928	−0.0350	0.130*	
C7	0.08495 (14)	0.5357 (7)	0.00033 (18)	0.0784 (12)	
C8	0.08172 (16)	0.4032 (8)	−0.03669 (19)	0.0912 (16)	
H8A	0.0905	0.4142	−0.0667	0.109*	
C9	0.06576 (19)	0.2613 (9)	−0.0279 (2)	0.1072 (19)	
H9A	0.0644	0.1735	−0.0516	0.129*	
C10	0.05146 (17)	0.2409 (8)	0.0143 (2)	0.1002 (16)	
H10A	0.0402	0.1408	0.0187	0.120*	
C11	0.05356 (14)	0.3666 (6)	0.05021 (18)	0.0737 (12)	
H11A	0.0433	0.3512	0.0786	0.088*	
C12	0.07062 (12)	0.5175 (6)	0.04549 (15)	0.0641 (11)	
C13	0.16592 (17)	0.5149 (4)	0.37445 (16)	0.0638 (11)	
C14	0.20119 (15)	0.5898 (5)	0.43349 (15)	0.0676 (12)	
H14A	0.1916	0.5664	0.4638	0.081*	
H14B	0.2317	0.5409	0.4460	0.081*	
C15	0.20519 (12)	0.7702 (4)	0.42897 (13)	0.0492 (9)	
C16	0.17724 (13)	0.8677 (6)	0.44192 (16)	0.0655 (11)	
H16A	0.1560	0.8203	0.4525	0.079*	
C17	0.17903 (17)	1.0338 (6)	0.44005 (19)	0.0818 (14)	
H17A	0.1594	1.0964	0.4495	0.098*	
C18	0.20899 (19)	1.1047 (5)	0.42461 (18)	0.0820 (14)	
H18A	0.2097	1.2170	0.4230	0.098*	
C19	0.23921 (14)	1.0145 (5)	0.41079 (15)	0.0629 (11)	
C20	0.2712 (2)	1.0871 (8)	0.3958 (2)	0.1019 (19)	
H20A	0.2722	1.1993	0.3938	0.122*	
C21	0.3005 (2)	0.9985 (12)	0.3841 (2)	0.116 (2)	
H21A	0.3211	1.0504	0.3734	0.140*	
C22	0.30117 (18)	0.8311 (11)	0.3874 (2)	0.111 (2)	
H22A	0.3229	0.7723	0.3807	0.133*	
C23	0.26923 (15)	0.7514 (7)	0.40089 (16)	0.0795 (13)	
H23A	0.2687	0.6390	0.4018	0.095*	
C24	0.23771 (12)	0.8422 (5)	0.41318 (13)	0.0525 (9)	
C25	0.05275 (12)	0.2135 (5)	0.32507 (14)	0.0537 (9)	
H25A	0.0477	0.3213	0.3308	0.064*	
C26	0.02851 (12)	0.0962 (5)	0.33832 (14)	0.0535 (9)	
C27	0.03634 (12)	−0.0623 (5)	0.32856 (15)	0.0577 (10)	
H27A	0.0202	−0.1448	0.3361	0.069*	
C28	0.06785 (12)	−0.0999 (4)	0.30777 (15)	0.0514 (9)	
H28A	0.0733	−0.2070	0.3015	0.062*	
C29	0.09120 (11)	0.0246 (4)	0.29648 (13)	0.0412 (7)	
C30	0.12614 (11)	−0.0026 (4)	0.27505 (13)	0.0401 (7)	
C31	0.14199 (12)	−0.1536 (4)	0.26986 (14)	0.0496 (8)	
H31A	0.1304	−0.2456	0.2795	0.060*	
C32	0.17511 (12)	−0.1673 (5)	0.25029 (15)	0.0571 (9)	
H32A	0.1859	−0.2690	0.2467	0.069*	
C33	0.19239 (13)	−0.0319 (5)	0.23602 (16)	0.0575 (9)	
C34	0.17446 (12)	0.1158 (4)	0.24173 (16)	0.0537 (9)	
H34A	0.1853	0.2089	0.2316	0.064*	
C35	−0.00465 (14)	0.1416 (6)	0.36237 (18)	0.0762 (12)	
H35A	−0.0321	0.0742	0.3444	0.114*	
H35B	−0.0137	0.2529	0.3532	0.114*	
H35C	0.0106	0.1267	0.4049	0.114*	
C36	0.22968 (16)	−0.0420 (6)	0.2159 (2)	0.0877 (14)	
H36A	0.2215	−0.1249	0.1862	0.131*	
H36B	0.2594	−0.0675	0.2495	0.131*	
H36C	0.2319	0.0601	0.1993	0.131*	
O5	0.0000	0.5928 (8)	0.2500	0.233 (5)	
H5	0.0203	0.5336	0.2496	0.350*	

### Atomic displacement parameters (Å^2^)

**Table:**

	*U*^11^	*U*^22^	*U*^33^	*U*^12^	*U*^13^	*U*^23^
Zn1	0.0513 (2)	0.0329 (2)	0.0490 (2)	−0.0034 (2)	0.01794 (17)	−0.00328 (19)
O1	0.0735 (16)	0.0611 (17)	0.0491 (13)	0.0086 (12)	0.0271 (12)	0.0098 (11)
O2	0.0709 (18)	0.0643 (18)	0.0627 (16)	0.0214 (15)	0.0243 (14)	0.0167 (14)
O3	0.0811 (19)	0.0525 (17)	0.0566 (15)	−0.0065 (14)	0.0180 (15)	−0.0072 (13)
O4	0.087 (2)	0.093 (2)	0.0689 (18)	−0.030 (2)	0.0246 (17)	−0.0223 (17)
N1	0.0496 (16)	0.0399 (17)	0.0419 (14)	0.0014 (13)	0.0189 (13)	−0.0009 (12)
N2	0.0438 (15)	0.0358 (16)	0.0497 (15)	−0.0030 (13)	0.0191 (13)	−0.0008 (13)
C1	0.073 (3)	0.039 (2)	0.0403 (19)	0.0021 (19)	0.0200 (19)	−0.0002 (16)
C2	0.073 (3)	0.064 (3)	0.057 (2)	0.021 (2)	0.027 (2)	0.014 (2)
C3	0.052 (2)	0.067 (3)	0.064 (2)	0.014 (2)	0.0175 (19)	0.033 (2)
C4	0.088 (3)	0.080 (4)	0.097 (4)	0.015 (3)	0.027 (3)	0.035 (3)
C5	0.091 (4)	0.102 (5)	0.125 (5)	0.003 (3)	0.048 (4)	0.065 (4)
C6	0.085 (4)	0.127 (6)	0.109 (4)	0.017 (4)	0.043 (3)	0.064 (4)
C7	0.059 (3)	0.112 (4)	0.061 (3)	0.021 (3)	0.026 (2)	0.028 (2)
C8	0.071 (3)	0.152 (5)	0.050 (2)	0.031 (3)	0.028 (2)	0.014 (3)
C9	0.095 (4)	0.149 (6)	0.066 (3)	0.017 (4)	0.028 (3)	−0.002 (4)
C10	0.090 (4)	0.114 (5)	0.083 (3)	−0.006 (3)	0.029 (3)	−0.014 (3)
C11	0.067 (3)	0.090 (3)	0.061 (2)	0.002 (3)	0.028 (2)	0.000 (3)
C12	0.045 (2)	0.102 (4)	0.042 (2)	0.018 (2)	0.0176 (17)	0.022 (2)
C13	0.091 (3)	0.035 (2)	0.042 (2)	−0.013 (2)	0.011 (2)	0.0039 (17)
C14	0.089 (3)	0.047 (2)	0.0400 (19)	−0.020 (2)	0.0077 (19)	−0.0006 (16)
C15	0.052 (2)	0.046 (2)	0.0334 (17)	−0.0076 (17)	0.0067 (16)	−0.0008 (15)
C16	0.055 (2)	0.077 (3)	0.051 (2)	−0.002 (2)	0.0142 (18)	−0.008 (2)
C17	0.075 (3)	0.074 (3)	0.073 (3)	0.021 (3)	0.014 (2)	−0.011 (3)
C18	0.099 (4)	0.044 (2)	0.061 (3)	0.008 (3)	0.003 (3)	0.003 (2)
C19	0.064 (3)	0.059 (3)	0.044 (2)	−0.019 (2)	0.0076 (19)	0.0078 (18)
C20	0.092 (4)	0.118 (5)	0.067 (3)	−0.045 (4)	0.013 (3)	0.015 (3)
C21	0.076 (4)	0.185 (8)	0.075 (4)	−0.045 (5)	0.025 (3)	0.006 (5)
C22	0.065 (3)	0.198 (8)	0.064 (3)	0.002 (4)	0.025 (2)	−0.019 (4)
C23	0.070 (3)	0.109 (4)	0.050 (2)	0.004 (3)	0.020 (2)	−0.015 (2)
C24	0.0480 (19)	0.064 (2)	0.0318 (16)	−0.0043 (19)	0.0071 (15)	−0.0030 (17)
C25	0.058 (2)	0.050 (2)	0.050 (2)	0.0059 (18)	0.0224 (18)	−0.0012 (17)
C26	0.049 (2)	0.064 (3)	0.0421 (19)	0.0022 (18)	0.0167 (17)	0.0041 (17)
C27	0.053 (2)	0.062 (3)	0.053 (2)	−0.0085 (19)	0.0199 (18)	0.0090 (19)
C28	0.052 (2)	0.040 (2)	0.058 (2)	−0.0038 (16)	0.0228 (18)	0.0003 (16)
C29	0.0428 (19)	0.0373 (18)	0.0340 (16)	−0.0004 (15)	0.0101 (14)	0.0013 (14)
C30	0.0405 (18)	0.0328 (17)	0.0363 (16)	−0.0014 (14)	0.0091 (14)	−0.0019 (14)
C31	0.057 (2)	0.0340 (18)	0.0529 (19)	−0.0014 (17)	0.0214 (17)	0.0010 (17)
C32	0.062 (2)	0.044 (2)	0.065 (2)	0.0070 (19)	0.0292 (19)	−0.0030 (18)
C33	0.060 (2)	0.053 (2)	0.064 (2)	0.0037 (19)	0.032 (2)	−0.0004 (19)
C34	0.055 (2)	0.043 (2)	0.066 (2)	−0.0055 (17)	0.0298 (19)	0.0020 (17)
C35	0.070 (3)	0.095 (3)	0.076 (3)	0.014 (3)	0.045 (2)	0.010 (3)
C36	0.098 (3)	0.076 (3)	0.122 (4)	0.015 (3)	0.079 (3)	0.010 (3)
O5	0.134 (5)	0.083 (5)	0.495 (15)	0.000	0.157 (8)	0.000

### Geometric parameters (Å, °)

**Table:**

Zn1—O1	1.967 (2)	C16—C17	1.376 (6)
Zn1—O3	2.009 (3)	C16—H16A	0.9300
Zn1—N2	2.072 (3)	C17—C18	1.334 (6)
Zn1—N1	2.098 (3)	C17—H17A	0.9300
Zn1—O4	2.416 (3)	C18—C19	1.394 (6)
O1—C1	1.273 (4)	C18—H18A	0.9300
O2—C1	1.224 (4)	C19—C20	1.391 (6)
O3—C13	1.258 (5)	C19—C24	1.427 (5)
O4—C13	1.240 (5)	C20—C21	1.333 (8)
N1—C29	1.344 (4)	C20—H20A	0.9300
N1—C25	1.340 (4)	C21—C22	1.386 (9)
N2—C34	1.334 (4)	C21—H21A	0.9300
N2—C30	1.348 (4)	C22—C23	1.392 (7)
C1—C2	1.520 (5)	C22—H22A	0.9300
C2—C3	1.507 (5)	C23—C24	1.410 (5)
C2—H2A	0.9700	C23—H23A	0.9300
C2—H2B	0.9700	C25—C26	1.381 (5)
C3—C4	1.363 (6)	C25—H25A	0.9300
C3—C12	1.437 (6)	C26—C27	1.378 (5)
C4—C5	1.470 (7)	C26—C35	1.503 (5)
C4—H4A	0.9300	C27—C28	1.379 (5)
C5—C6	1.383 (8)	C27—H27A	0.9300
C5—H5A	0.9300	C28—C29	1.381 (4)
C6—C7	1.352 (7)	C28—H28A	0.9300
C6—H6A	0.9300	C29—C30	1.477 (4)
C7—C8	1.414 (7)	C30—C31	1.378 (4)
C7—C12	1.427 (5)	C31—C32	1.375 (5)
C8—C9	1.340 (8)	C31—H31A	0.9300
C8—H8A	0.9300	C32—C33	1.372 (5)
C9—C10	1.356 (7)	C32—H32A	0.9300
C9—H9A	0.9300	C33—C34	1.387 (5)
C10—C11	1.362 (6)	C33—C36	1.509 (5)
C10—H10A	0.9300	C34—H34A	0.9300
C11—C12	1.391 (6)	C35—H35A	0.9600
C11—H11A	0.9300	C35—H35B	0.9600
C13—C14	1.523 (5)	C35—H35C	0.9600
C14—C15	1.505 (5)	C36—H36A	0.9600
C14—H14A	0.9700	C36—H36B	0.9600
C14—H14B	0.9700	C36—H36C	0.9600
C15—C16	1.358 (5)	O5—H5	0.8199
C15—C24	1.415 (5)		
			
O1—Zn1—O3	121.37 (11)	C15—C16—C17	122.7 (4)
O1—Zn1—N2	120.53 (10)	C15—C16—H16A	118.6
O3—Zn1—N2	103.04 (11)	C17—C16—H16A	118.6
O1—Zn1—N1	104.43 (11)	C18—C17—C16	119.7 (5)
O3—Zn1—N1	122.09 (10)	C18—C17—H17A	120.1
N2—Zn1—N1	78.64 (11)	C16—C17—H17A	120.1
O1—Zn1—O4	93.91 (11)	C17—C18—C19	121.6 (4)
O3—Zn1—O4	58.10 (11)	C17—C18—H18A	119.2
N2—Zn1—O4	145.01 (11)	C19—C18—H18A	119.2
N1—Zn1—O4	87.49 (11)	C20—C19—C18	122.1 (5)
C1—O1—Zn1	104.8 (2)	C20—C19—C24	119.1 (5)
C13—O3—Zn1	99.2 (3)	C18—C19—C24	118.8 (4)
C13—O4—Zn1	80.9 (2)	C21—C20—C19	121.1 (6)
C29—N1—C25	118.7 (3)	C21—C20—H20A	119.5
C29—N1—Zn1	114.6 (2)	C19—C20—H20A	119.5
C25—N1—Zn1	126.6 (2)	C20—C21—C22	121.8 (6)
C34—N2—C30	118.6 (3)	C20—C21—H21A	119.1
C34—N2—Zn1	125.8 (2)	C22—C21—H21A	119.1
C30—N2—Zn1	115.3 (2)	C21—C22—C23	119.8 (6)
O2—C1—O1	122.7 (3)	C21—C22—H22A	120.1
O2—C1—C2	122.0 (3)	C23—C22—H22A	120.1
O1—C1—C2	115.4 (3)	C22—C23—C24	119.5 (5)
C3—C2—C1	115.9 (3)	C22—C23—H23A	120.2
C3—C2—H2A	108.3	C24—C23—H23A	120.2
C1—C2—H2A	108.3	C23—C24—C15	122.9 (4)
C3—C2—H2B	108.3	C23—C24—C19	118.7 (4)
C1—C2—H2B	108.3	C15—C24—C19	118.4 (4)
H2A—C2—H2B	107.4	N1—C25—C26	123.5 (3)
C4—C3—C12	119.1 (4)	N1—C25—H25A	118.3
C4—C3—C2	121.5 (5)	C26—C25—H25A	118.3
C12—C3—C2	119.4 (4)	C27—C26—C25	116.9 (3)
C3—C4—C5	120.6 (5)	C27—C26—C35	122.3 (4)
C3—C4—H4A	119.7	C25—C26—C35	120.8 (4)
C5—C4—H4A	119.7	C26—C27—C28	120.8 (4)
C6—C5—C4	117.2 (5)	C26—C27—H27A	119.6
C6—C5—H5A	121.4	C28—C27—H27A	119.6
C4—C5—H5A	121.4	C27—C28—C29	118.7 (3)
C7—C6—C5	124.4 (6)	C27—C28—H28A	120.7
C7—C6—H6A	117.8	C29—C28—H28A	120.7
C5—C6—H6A	117.8	N1—C29—C28	121.4 (3)
C6—C7—C8	122.0 (5)	N1—C29—C30	115.6 (3)
C6—C7—C12	118.1 (5)	C28—C29—C30	123.0 (3)
C8—C7—C12	119.9 (5)	N2—C30—C31	120.9 (3)
C9—C8—C7	118.9 (5)	N2—C30—C29	115.6 (3)
C9—C8—H8A	120.6	C31—C30—C29	123.5 (3)
C7—C8—H8A	120.6	C32—C31—C30	119.6 (3)
C8—C9—C10	122.6 (6)	C32—C31—H31A	120.2
C8—C9—H9A	118.7	C30—C31—H31A	120.2
C10—C9—H9A	118.7	C31—C32—C33	120.4 (4)
C9—C10—C11	120.1 (6)	C31—C32—H32A	119.8
C9—C10—H10A	120.0	C33—C32—H32A	119.8
C11—C10—H10A	120.0	C32—C33—C34	116.8 (3)
C10—C11—C12	121.7 (5)	C32—C33—C36	121.9 (4)
C10—C11—H11A	119.1	C34—C33—C36	121.3 (4)
C12—C11—H11A	119.1	N2—C34—C33	123.8 (3)
C11—C12—C7	116.9 (5)	N2—C34—H34A	118.1
C11—C12—C3	122.5 (4)	C33—C34—H34A	118.1
C7—C12—C3	120.6 (4)	C26—C35—H35A	109.5
O4—C13—O3	121.4 (4)	C26—C35—H35B	109.5
O4—C13—C14	119.6 (4)	H35A—C35—H35B	109.5
O3—C13—C14	119.1 (4)	C26—C35—H35C	109.5
C15—C14—C13	112.3 (3)	H35A—C35—H35C	109.5
C15—C14—H14A	109.1	H35B—C35—H35C	109.5
C13—C14—H14A	109.1	C33—C36—H36A	109.5
C15—C14—H14B	109.1	C33—C36—H36B	109.5
C13—C14—H14B	109.1	H36A—C36—H36B	109.5
H14A—C14—H14B	107.9	C33—C36—H36C	109.5
C16—C15—C24	118.7 (4)	H36A—C36—H36C	109.5
C16—C15—C14	118.7 (4)	H36B—C36—H36C	109.5
C24—C15—C14	122.6 (4)		
			
O3—Zn1—O1—C1	69.3 (3)	Zn1—O3—C13—O4	−7.8 (4)
N2—Zn1—O1—C1	−62.5 (3)	Zn1—O3—C13—C14	171.6 (3)
N1—Zn1—O1—C1	−147.6 (2)	O4—C13—C14—C15	−81.9 (5)
O4—Zn1—O1—C1	124.0 (2)	O3—C13—C14—C15	98.7 (4)
O1—Zn1—O3—C13	77.4 (2)	C13—C14—C15—C16	91.5 (4)
N2—Zn1—O3—C13	−143.8 (2)	C13—C14—C15—C24	−90.0 (5)
N1—Zn1—O3—C13	−59.2 (3)	C24—C15—C16—C17	0.3 (5)
O4—Zn1—O3—C13	4.0 (2)	C14—C15—C16—C17	178.8 (3)
O1—Zn1—O4—C13	−128.9 (2)	C15—C16—C17—C18	0.5 (6)
O3—Zn1—O4—C13	−4.1 (2)	C16—C17—C18—C19	−0.7 (6)
N2—Zn1—O4—C13	60.8 (3)	C17—C18—C19—C20	−178.7 (4)
N1—Zn1—O4—C13	126.8 (2)	C17—C18—C19—C24	0.2 (6)
O1—Zn1—N1—C29	119.4 (2)	C18—C19—C20—C21	178.4 (4)
O3—Zn1—N1—C29	−97.9 (2)	C24—C19—C20—C21	−0.5 (6)
N2—Zn1—N1—C29	0.5 (2)	C19—C20—C21—C22	−1.2 (8)
O4—Zn1—N1—C29	−147.2 (2)	C20—C21—C22—C23	2.7 (8)
O1—Zn1—N1—C25	−59.6 (3)	C21—C22—C23—C24	−2.4 (7)
O3—Zn1—N1—C25	83.1 (3)	C22—C23—C24—C15	−178.1 (3)
N2—Zn1—N1—C25	−178.5 (3)	C22—C23—C24—C19	0.7 (5)
O4—Zn1—N1—C25	33.8 (3)	C16—C15—C24—C23	177.9 (3)
O1—Zn1—N2—C34	83.3 (3)	C14—C15—C24—C23	−0.5 (5)
O3—Zn1—N2—C34	−55.9 (3)	C16—C15—C24—C19	−0.8 (4)
N1—Zn1—N2—C34	−176.6 (3)	C14—C15—C24—C19	−179.3 (3)
O4—Zn1—N2—C34	−108.0 (3)	C20—C19—C24—C23	0.8 (5)
O1—Zn1—N2—C30	−103.9 (2)	C18—C19—C24—C23	−178.2 (3)
O3—Zn1—N2—C30	116.9 (2)	C20—C19—C24—C15	179.5 (3)
N1—Zn1—N2—C30	−3.7 (2)	C18—C19—C24—C15	0.6 (5)
O4—Zn1—N2—C30	64.9 (3)	C29—N1—C25—C26	−0.7 (5)
Zn1—O1—C1—O2	1.7 (4)	Zn1—N1—C25—C26	178.3 (2)
Zn1—O1—C1—C2	−176.5 (3)	N1—C25—C26—C27	−0.8 (5)
O2—C1—C2—C3	12.7 (6)	N1—C25—C26—C35	179.0 (3)
O1—C1—C2—C3	−169.0 (3)	C25—C26—C27—C28	1.4 (5)
C1—C2—C3—C4	−110.8 (4)	C35—C26—C27—C28	−178.5 (3)
C1—C2—C3—C12	69.5 (5)	C26—C27—C28—C29	−0.5 (5)
C12—C3—C4—C5	0.6 (6)	C25—N1—C29—C28	1.7 (4)
C2—C3—C4—C5	−179.2 (4)	Zn1—N1—C29—C28	−177.4 (2)
C3—C4—C5—C6	−0.4 (7)	C25—N1—C29—C30	−178.4 (3)
C4—C5—C6—C7	−0.3 (8)	Zn1—N1—C29—C30	2.5 (3)
C5—C6—C7—C8	−178.9 (5)	C27—C28—C29—N1	−1.1 (5)
C5—C6—C7—C12	0.7 (8)	C27—C28—C29—C30	179.0 (3)
C6—C7—C8—C9	178.6 (5)	C34—N2—C30—C31	0.1 (4)
C12—C7—C8—C9	−1.0 (7)	Zn1—N2—C30—C31	−173.3 (2)
C7—C8—C9—C10	1.7 (8)	C34—N2—C30—C29	179.5 (3)
C8—C9—C10—C11	−0.8 (8)	Zn1—N2—C30—C29	6.1 (3)
C9—C10—C11—C12	−0.7 (7)	N1—C29—C30—N2	−5.7 (4)
C10—C11—C12—C7	1.3 (6)	C28—C29—C30—N2	174.1 (3)
C10—C11—C12—C3	−178.2 (4)	N1—C29—C30—C31	173.7 (3)
C6—C7—C12—C11	180.0 (4)	C28—C29—C30—C31	−6.5 (5)
C8—C7—C12—C11	−0.4 (6)	N2—C30—C31—C32	0.3 (5)
C6—C7—C12—C3	−0.5 (6)	C29—C30—C31—C32	−179.1 (3)
C8—C7—C12—C3	179.1 (4)	C30—C31—C32—C33	0.0 (5)
C4—C3—C12—C11	179.4 (4)	C31—C32—C33—C34	−0.7 (5)
C2—C3—C12—C11	−0.9 (5)	C31—C32—C33—C36	178.4 (4)
C4—C3—C12—C7	−0.1 (6)	C30—N2—C34—C33	−0.8 (5)
C2—C3—C12—C7	179.6 (3)	Zn1—N2—C34—C33	171.8 (3)
Zn1—O4—C13—O3	6.5 (3)	C32—C33—C34—N2	1.1 (5)
Zn1—O4—C13—C14	−172.9 (3)	C36—C33—C34—N2	−177.9 (3)

### Hydrogen-bond geometry (Å, °)

**Table:**

*D*—H···*A*	*D*—H	H···*A*	*D*···*A*	*D*—H···*A*
O5—H5···O1	0.82	2.21	2.948 (3)	150
C8—H8A···O4^i^	0.93	2.59	3.515 (6)	171

Symmetry codes: (i) *x*, −*y*+1, *z*−1/2.

## References

[bb1] Bruker (2004). *APEX2* and *SAINT* Bruker AXS Inc., Madison, Wisconsin, USA.

[bb2] Guilera, G. & Steed, J. W. (1999). *Chem. Commun.* **6**, 1294–1296.

[bb3] Kong, Z. G., Wang, X. Y. & Carlucci, L. (2009). *Inorg. Chem. Commun.* **12**, 691–694

[bb4] Li, Y.-P., Sun, D.-J., Zang, H., Su, G.-F. & Li, Y.-L. (2009). *Acta Cryst.* C**65**, m340–m342. 10.1107/S0108270109029084 19726847

[bb5] Sheldrick, G. M. (1996). *SADABS* University of Göttingen, Germany.

[bb6] Sheldrick, G. M. (2008). *Acta Cryst.* A**64**, 112–122. 10.1107/S0108767307043930 18156677

